# Isolation and characterization of indigenous probiotic bacteria from wild Nile tilapia (*Oreochromis niloticus*) in Lake Naivasha, Kenya, with first evidence of *Paucilactobacillus vaccinostercus* as a potential aquaculture probiotic

**DOI:** 10.14202/vetworld.2026.805-820

**Published:** 2026-02-28

**Authors:** Rosaline D. Karimi, Daniel W. Wanja, Joseph J.N. Ngeranwa, Philip N. Nyaga

**Affiliations:** 1 Department of Biochemistry and Biotechnology, School of Pure and Applied Science, Kenyatta University, P.O. Box 43844-00100 Nairobi, Kenya; 2 Kenya Fisheries Service, NHIF Building, P.O. Box 48511-00100, Nairobi, Kenya; 3 Department of Veterinary Pathology, Microbiology and Parasitology, Faculty of Veterinary Medicine and Surgery, Egerton University, P.O. Box 536-20115, Egerton, Kenya; 4 Department of Veterinary Pathology, Microbiology and Parasitology, Faculty of Veterinary Medicine, University of Nairobi, P.O. Box 29053-00625, Kangemi, Nairobi, Kenya

**Keywords:** aquaculture, aquaculture probiotics, fish gut microbiota, Kenya aquaculture, Lake Naivasha, MALDI-TOF MS, Nile tilapia, *Paucilactobacillus vaccinostercus*, sustainable aquaculture

## Abstract

**Background and Aim:**

The growing burden of infectious diseases and antimicrobial resistance (AMR) in aquaculture demands safe, host-adapted alternatives to antibiotics. Probiotics derived from the gastrointestinal tract (GIT) of the target host are considered more ecologically compatible and effective than non-host strains. This study aimed to isolate and characterize indigenous gut bacteria from wild Nile tilapia (*Oreochromis niloticus*) collected from Lake Naivasha, Kenya, to evaluate their probiotic attributes, assess pathogenicity, and identify promising candidates using conventional phenotypic methods and matrix-assisted laser desorption/ionization time-of-flight mass spectrometry (MALDI-TOF MS).

**Materials and Methods:**

Thirty-eight apparently healthy *O. niloticus* were sampled, and bacteria were isolated from the entire GIT using standard bacteriological techniques. Fifty autochthonous isolates were obtained and subjected to stepwise screening, including hemolytic activity, antibiotic susceptibility, enzymatic activity (protease and amylase), tolerance to low pH and bile salts, adhesion to stainless steel surfaces, growth kinetics, and *in vivo* pathogenicity in *O. niloticus*. Species-level identification was performed using biochemical tests and MALDI-TOF MS.

**Results:**

Of the 50 isolates, 10 (20%) were nonhemolytic and sensitive to at least eight antibiotics. Functional screening reduced these to four candidates exhibiting enzymatic activity, acid and bile tolerance, and adhesion. Three isolates, identified as *Rossellomorea marisflavi*, *Micrococcus luteus*, and *Paucilactobacillus vaccinostercus*, were nonpathogenic to *O. niloticus*. In contrast, *Aeromonas ichthiosmia*, despite exhibiting several probiotic-like traits *in vitro*, caused 80% cumulative mortality and was excluded. Among the nonpathogenic isolates, *P. vaccinostercus* demonstrated the strongest overall probiotic profile, including superior acid and bile tolerance, high enzymatic indices, robust adhesion (~4.7 × 10^4^ CFU/mL), and favorable growth kinetics.

**Conclusion:**

The gut microbiota of wild *O. niloticus* from Lake Naivasha harbors a limited but valuable pool of indigenous bacteria with probiotic potential. This study provides the first evidence that *P. vaccinostercus* is a promising, nonpathogenic probiotic candidate for tilapia aquaculture. These findings support the development of locally adapted, antibiotic-free probiotic strategies to enhance fish health and sustainable aquaculture in Kenya. Further *in vivo* feeding trials and genomic safety assessments are warranted.

## INTRODUCTION

Aquaculture is one of the most rapidly expanding food production sectors globally; however, disease outbreaks continue to constrain productivity [1]. The aquaculture sector has substantial potential to contribute to the attainment of Vision 2030, with an anticipated annual economic growth rate of 10%. At both innovative and commercial scales, aquaculture production is expected to enhance food security, generate employment and wealth, increase revenue, and support national development. Nile tilapia (*Oreochromis niloticus*), a key aquaculture species in Africa, is widely cultivated for its high adaptability and economic importance [2]. Production of *O. niloticus* has quadrupled over the past decade, largely because it thrives under diverse physical and environmental conditions. This species reproduces readily in captivity and shows relatively high tolerance to handling stress and pathogens compared with other cultured fish. Its strong market demand and stable pricing further reinforce its suitability for aquaculture production. Tilapia is increasingly recognized as the preferred species for intensive aquaculture and is projected to become the most important cultured fish globally [3]. Nevertheless, despite its considerable potential, tilapia farming remains highly vulnerable to diseases [4] and suboptimal water quality [5].

Globally, viral, bacterial, and fungal infections have caused devastating economic losses in aquaculture. Bacterial diseases are a major threat, particularly in farmed tilapia and catfish [4]. Several pathogenic bacteria, including *Aeromonas hydrophila*, *Aeromonas veronii*, *Acinetobacter* spp., *Vibrio parahaemolyticus*, *Pseudomonas fluorescens*, *Edwardsiella tarda*, *Flavobacterium columnare*, and *Streptococcus iniae*, have been implicated in disease outbreaks in Kenyan waters [6–11]. These infections commonly present as fin rot, ulcers, exophthalmia, and abdominal distension, conditions that are frequently exacerbated by stress and poor water quality [10]. Consequently, antimicrobial agents have been widely used to control bacterial diseases in aquaculture, contributing to the emergence of antimicrobial resistance (AMR) [12, 13]. Antimicrobial use may also result in drug residues in fish products, leading to market restrictions and potential risks to public health and the environment. Concerns about AMR and antimicrobial residues in fish products have been reported in Kenya and other parts of sub-Saharan Africa [14, 15]. Recent surveillance studies in selected Kenyan counties have further identified opportunistic pathogens, including *Aeromonas*, *Pseudomonas*, *Citrobacter*, *Streptococcus*, *Escherichia*, *Proteus*, and *Flavobacterium*, with many strains exhibiting multidrug resistance, particularly to ampicillin and cotrimoxazole, while remaining susceptible to gentamicin and selected disinfectants [6–8]. The public health risks associated with AMR and antimicrobial residues underscore the urgent need for safer alternatives to antibiotics in aquaculture [16, 17]. Probiotics, prebiotics, and synbiotics offer promising strategies to reduce pathogen load and enhance disease resistance without promoting AMR [18, 19].

Probiotics, defined as live microorganisms that confer health benefits on the host when administered in adequate amounts, have shown potential to improve digestion, enhance immune responses, and increase resistance to pathogens in fish [20, 21]. Hossain *et al*. [22] described probiotics as live microbial feed supplements that improve intestinal microbial balance in the host. Most probiotics are bacterial, with lactic acid bacteria (LAB) being the most commonly used; however, certain molds and yeasts are also employed [23]. Probiotic candidates reported in aquaculture include species of *Bacillus*, *Alteromonas*, *Arthrobacter*, *Bifidobacterium*, *Clostridium*, *Paenibacillus*, *Phaeobacter*, *Pseudoalteromonas*, *Pseudomonas*, *Rhodosporidium*, *Roseobacter*, *Carnobacterium*, *Enterococcus*, *Streptococcus*, *Pediococcus*, *Propionibacterium*, *Leuconostoc*, *Lactobacillus*, *Lactococcus*, and *Streptomyces*, as well as microalgae (*Tetraselmis*), yeasts from the genera *Debaryomyces*, *Phaffia*, and *Saccharomyces*, and molds such as *Aspergillus* [21, 24]. In addition, some isolates from pathogenic genera, including *Aeromonas* and *Vibrio*, have exhibited probiotic properties [25, 26]. The application of probiotics can mitigate production constraints by improving fish growth and nutritional efficiency, addressing challenges associated with stunted growth and limited adoption of modern production practices [27]. El-Kady *et al*. [28] further demonstrated that probiotics enhance disease resistance, growth performance, and water quality in aquaculture systems.

The fish gastrointestinal tract (GIT) serves as a natural reservoir for potential probiotic bacteria [29]. Probiotics currently used in aquaculture are largely derived from non-piscine sources and may therefore fail to elicit optimal host-specific responses in aquatic species [30]. Although commercial probiotic products are available, native bacteria isolated from the host fish species are considered the most effective dietary probiotic supplements [31].

Despite growing interest in probiotic-based interventions to reduce disease burden and AMR in aquaculture, critical gaps remain in identifying and validating host-adapted probiotic strains for tilapia farming in East Africa. Most probiotics currently applied in aquaculture are derived from non-piscine or non-native sources and may therefore exhibit limited colonization efficiency, ecological compatibility, and functional performance within the GIT of target fish species. In Kenya, existing studies have largely focused on pond-reared fish, commercial probiotic formulations, or pathogen surveillance, with minimal emphasis on the systematic isolation and functional screening of indigenous gut microbiota from wild fish populations. Consequently, baseline data on the diversity, safety, and probiotic potential of autochthonous gut bacteria in wild *O. niloticus* from natural freshwater ecosystems are lacking. Moreover, many probiotic screening studies rely solely on *in vitro* assays, omitting pathogenicity testing, thereby creating uncertainty about host safety. The limited application of advanced identification tools, such as matrix-assisted laser desorption/ionization time-of-flight mass spectrometry (MALDI-TOF MS), further limits accurate species-level characterization of candidate probiotics in local aquaculture research. These gaps hinder the development of locally adapted, evidence-based probiotic strategies that align with sustainable aquaculture, One Health, and AMR mitigation goals.

The present study aimed to address these gaps by isolating indigenous gut bacteria from wild *O. niloticus* inhabiting Lake Naivasha and systematically evaluating their probiotic potential. Specifically, the study aimed to assess the isolates for key probiotic attributes, including enzymatic activity, tolerance to acid and bile salts, adhesion capability, growth kinetics, and antibiotic susceptibility, while concurrently evaluating their pathogenicity to *O. niloticus*. In addition, the study sought to accurately identify promising probiotic candidates using conventional phenotypic methods and MALDI-TOF MS. By focusing on host-derived, nonpathogenic, and functionally robust bacterial strains, this study aimed to generate foundational evidence for the development of ecologically compatible probiotic supplements that could enhance fish health, reduce reliance on antimicrobials, and support sustainable tilapia aquaculture in Kenya.

## MATERIALS AND METHODS

### Ethical approval

Ethical approval (FVM BAUEC/2019/193) and a research permit (NACOSTI/P/18/64308/21246) were obtained from the Faculty of Veterinary Medicine Biosafety, Animal Use and Ethical Committee, University of Nairobi, and from the National Commission for Science, Technology and Innovations (NACOSTI), respectively, prior to study commencement. Informed verbal consent to conduct the research was obtained from the Regional Director of Fisheries. All experimental procedures involving *O. niloticus* were performed in accordance with internationally accepted guidelines for the care and use of animals, and the Animal Research: Reporting of *In Vivo* Experiments 2.0 guidelines were strictly followed for study design, fish handling, and reporting [32].

### Study period and location

The study was conducted between January 2023 and November 2024 along the shorelines of Lake Naivasha, specifically at the Karagita Landing Beach. This landing beach was deliberately selected because of declining fish stocks reported at other locations within the lake.

**Figure 1:**
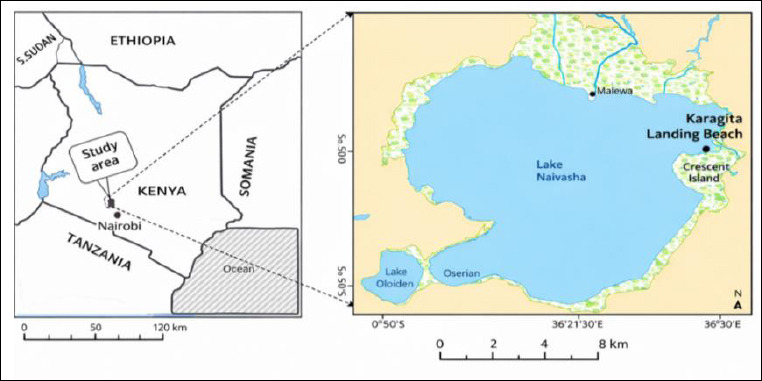
Geographical location of Lake Naivasha in Kenya and the specific sampling site along the lake shoreline. The map was generated using the Google Maps API and modified from Adhiambo et al. [35].

The lake supports high aquatic biodiversity and hosts several fish species, including blue-spotted tilapia (*Oreochromis leucostictus*), red-bellied tilapia (*Coptodon zillii*), largemouth bass (*Micropterus salmoides*), Louisiana red swamp crayfish (*Procambarus clarkii*), river cyprinid (*Barbus paludinosus*), common carp (*Cyprinus carpio*), *O. niloticus*, and African sharptooth catfish (*Clarias gariepinus*) [33]. Lake Naivasha is the second-largest freshwater lake in Kenya after the Kenyan portion of Lake Victoria [33]. It has a surface area of 139 km² and a mean depth of 3.35 m, with a maximum depth of 7 m; however, these parameters fluctuate under extreme hydrological conditions [34]. Naivasha town is situated approximately 80 km northwest of Nairobi, and the lake basin covers an area of ~3400 km². Lake Naivasha is located in the Eastern Rift Valley at a latitude of 0°46′10″ (0.7694), a longitude of 36°20′25″ (36.3403), and an altitude of 1890 m above sea level (Figure 1) [35].

### Fish inclusion and exclusion criteria and sampling

Fish included in this study were apparently healthy at the time of capture and free from visible external lesions or parasitic infestations. Individuals exhibiting skin lesions, fin rot, or external parasite infestations were excluded. Any exclusions made after capture were documented, and such fish were excluded post-capture.

A total of 38 table-sized *O. niloticus* (18 males and 20 females) meeting the inclusion criteria were collected. The fish had a mean body weight of 418.6 ± 39.0 g, standard length of 20.9 ± 0.8 cm, and total length of 25.8 ± 0.9 cm. Fish were randomly captured using seine nets attached to motorized boats at Karagita Landing Beach between 0700 and 0800 h in January 2023. Water quality parameters were not assessed at capture because sampling covered large portions of the lake; however, January typically corresponds to the hot–dry season.

Following capture, fish were placed in two separate 100-L plastic tanks containing source water and transported alive to the Bacteriology Laboratory, Department of Veterinary Pathology, Microbiology, and Parasitology, University of Nairobi. Laboratory analyses commenced within 2 h of arrival.

### Necropsy and bacterial isolation from the gut

Prior to necropsy, fish were humanely anesthetized using tricaine methane sulfonate (Syncaine®, Abbott Laboratories, Chicago, IL, USA) and euthanized in accordance with institutional animal-care guidelines. Postmortem procedures were conducted under aseptic conditions following standardized protocols described by Noga [36] and Roberts [37]. Dissecting instruments and bench surfaces were sterilized between samples using flaming and 70% ethanol, respectively, and gloves were changed between handling individual fish to minimize contamination. Separate cutting sets were used for each fish, and the necropsy sequence was standardized from external surfaces to internal organs to prevent microbial carryover.

Before opening the body cavity, fish skin surfaces were swabbed with 70% ethanol. Each fish underwent external examination, and gross lesions and biodata were recorded. A midline incision was made from the vent to the operculum, followed by a lateral incision along the abdominal wall to expose the viscera. The esophagus and rectum were severed, and the entire gut was removed. The hepatopancreas and mesentery were bluntly dissected and discarded. The gut was collected in sterile Petri dishes for bacterial isolation.

Field and laboratory blanks were included by processing phosphate-buffered saline (PBS; pH 7.2) alongside gut samples as negative controls to monitor environmental and procedural contamination.

Up to 25 g of gut tissue and contents were aseptically weighed and homogenized with 225 mL buffered peptone water to obtain an initial 1:10 dilution using a stomacher blender. The homogenate was serially diluted, and 0.1 mL aliquots of selected dilutions were inoculated onto tryptone soya agar (TSA; HiMedia Laboratories Pvt. Ltd., Mumbai, India) in duplicate and incubated aerobically at 24°C–25°C. After 24 h, plates were examined for growth and colony morphology. Single colonies were randomly selected, subcultured on TSA, and purified by repeated streaking. Pure isolates were transferred to tryptone soya broth (TSB; HiMedia) supplemented with 20% glycerol and stored at −80°C. Recovery was confirmed by thawing selected isolates and assessing growth on TSA.

### Preliminary screening of potential probiotic bacteria

Following the isolation of 50 bacterial strains, preliminary screening was performed. Hemolytic activity was assessed by streaking isolates onto 5% sheep blood agar and incubating aerobically at 24°C–25°C for 24 h. Hemolysis was classified as α, β, δ, or γ. Isolates exhibiting γ or α hemolysis were selected for further analyses.

Antibiotic susceptibility testing was performed using the Kirby–Bauer disk diffusion method in accordance with Clinical and Laboratory Standards Institute guidelines [38]. Bacterial suspensions were adjusted to a 0.5 McFarland standard (~1.5 × 10^8^ CFU/mL) and spread onto Mueller–Hinton agar (Oxoid Ltd., Basingstoke, UK). Antibiotic disks (HiMedia) included ampicillin, tetracycline, streptomycin, sulfonamides, nalidixic acid, trimethoprim–sulfamethoxazole, gentamicin, nitrofurantoin, chloramphenicol, and kanamycin. Inhibition zones were measured after 24 h and interpreted as sensitive or resistant as described by Patel *et al*. [39]. *Escherichia coli* American Type Culture Collection [ATCC]® 25922 and *Staphylococcus aureus* ATCC® 25923 were used as quality control strains.

### Functional screening of probiotic attributes

#### Proteolytic and amylolytic activities

Proteolytic and amylolytic activities were evaluated by inoculating isolates onto skim milk agar and starch agar (HiMedia), respectively [40]. Plates were incubated aerobically at 24°C–25°C for 48 h. Starch degradation was visualized using 1% Lugol’s iodine solution. Clear zones indicated enzymatic activity, and activity indices were calculated as described previously [41]. *Bacillus subtilis* ATCC 6051 and *Bacillus amyloliquefaciens* ATCC 23350 were used as positive controls, and *E. coli* ATCC 25922 served as a negative control.

#### Bile salt tolerance

Bile tolerance was assessed using bile salts (Sigma-Aldrich, St. Louis, MO, USA) incorporated into TSB (HiMedia) at concentrations of 0.3% and 2% following Govindaraj *et al*. [42]. TSB without bile served as the control. Viable counts were determined after incubation, and survival percentages were calculated. *Lactobacillus acidophilus* ATCC 4356 and *E. coli* ATCC 25922 were used as positive and negative controls, respectively.

#### Acid tolerance

Acid tolerance was assessed by exposing isolates to PBS adjusted to pH 1.5, 3.0, and 7.2 using 0.1 M HCl, as described by Reda *et al*. [40] and Govindaraj *et al*. [42]. Viable counts were determined at 0, 1.5, and 3 h. Survival percentages were calculated, and isolates with ≥ 40% survival were considered acid-tolerant.

#### Bacterial adhesion assay

Bacterial adhesion was evaluated using stainless steel plates (1 × 1 cm) as described by Mulyasari *et al*. [41]. Plates were incubated with bacterial suspensions in TSB, rinsed to remove non-adherent cells, and adherent bacteria were quantified by plate counting on TSA. *Lactobacillus plantarum* ATCC 14917 and sterile TSB served as positive and negative controls.

#### Bacterial growth kinetics

Growth kinetics were assessed by monitoring optical density at 600 nm at 1-h intervals for 9 h in TSB using a spectrophotometer, as described by Zhang *et al*. [43]. *L. plantarum* ATCC 14917 served as the positive control.

### Pathogenicity assessment in *O. niloticus*

Pathogenicity was evaluated based on cumulative mortality, clinical signs, and bacterial re-isolation. Sample size estimation was conducted using G*Power version 3.1.9.6 [44]. A total of 150 healthy *O. niloticus* were acclimatized and randomly assigned to treatment and control groups. Fish received intraperitoneal injections of bacterial suspensions or PBS (control). Fish were monitored for 10 days, and bacteria were re-isolated from spleen and kidney of moribund or dead fish. All challenge experiments were conducted under biosafety level 2 conditions.

### Identification of candidate probiotic bacteria

Candidate isolates were identified based on colony morphology, Gram staining, biochemical tests, and matrix-assisted laser desorption/ionization time-of-flight mass spectrometry (Bruker Daltonics GmbH, Bremen, Germany), following manufacturer criteria [45, 46].

### Statistical analysis

Enzymatic activity indices were calculated as described previously [41]. Bacterial counts were log_10-_ transformed, and data were expressed as mean ± SD. Normality and homogeneity were assessed using Shapiro–Wilk and Levene’s tests. Differences among groups were analyzed using chi-square tests and one-way analysis of variance, followed by Tukey’s post hoc test, with statistical significance set at p < 0.05. Analyses were performed using IBM SPSS Statistics (IBM Corp., Armonk, NY, USA), version 31.

## RESULTS

Preliminary screening based on hemolytic activity and antimicrobial susceptibility

A total of 50 bacterial isolates with distinct colonial morphologies were recovered from the gut of *O. niloticus*. Of these, only 10 isolates (20%) exhibited γ-hemolysis on blood agar and were therefore considered non-hemolytic. These isolates demonstrated sensitivity to at least eight antibiotics. Notably, isolates E, H, and I were susceptible to all 10 antibiotics tested (Table 1).

**Table 1:** Inhibition zone diameters (mm) and antimicrobial susceptibility profiles of the 10 bacterial isolates (A–J) against selected antibiotics based on the interpretative guidelines described by Patel *et al.* [39].

Antibiotic disk	Disk concentration	A	B	C	D	E	F	G	H	J	I
Ampicillin	10 μg	22 S	20 S	10 S	10 S	26 S	38 S	06 R	18 S	15 S	14 S
Tetracycline	30 μg	24 S	38 S	23 S	27 S	28 S	40 S	27 S	30 S	24 S	26 S
Streptomycin	10 μg	17 S	20 S	06 R	11 S	22 S	34 S	11 S	26 S	22 S	20 S
Sulfonamides	300 μg	25 S	30 S	30 S	28 S	40 S	40 S	25 S	40 S	23 S	16 S
Nalidixic acid	30 μg	30 S	13 S	26 S	28 S	22 S	06 R	28 S	12 S	12 S	15 S
Trimethoprim–sulfamethoxazole	1.25/23.75 μg	28 S	34 S	26 S	26 S	38 S	36 S	30 S	38 S	24 S	23 S
Gentamicin	10 μg	20 S	10 S	10 S	10 S	26 S	36 S	22 S	28 S	22 S	14 S
Nitrofurantoin	300 μg	18 S	06 R	24 S	10 S	24 S	06 R	24 S	23 S	15 S	21 S
Chloramphenicol	30 μg	09 R	09 R	10 S	09 R	10 S	11 S	10 S	11 S	09 R	10 S
Kanamycin	30 μg	13 S	15 S	06 R	11 S	12 S	12 S	10 S	18 S	14 S	12 S

A–J = Bacterial isolates identified in this study, S = Susceptible, R = Resistant.

Isolates A, B, D, and J exhibited resistance to chloramphenicol. Isolate B additionally showed resistance to nitrofurantoin. Isolates C and G were resistant to streptomycin and ampicillin, respectively. Isolate F displayed resistance to nalidixic acid and NF. Based on these findings, the 10 non-hemolytic and broadly susceptible isolates were advanced to functional *in vitro* screening.

### Proteolytic and amylolytic enzyme activities

**Figure 2:**
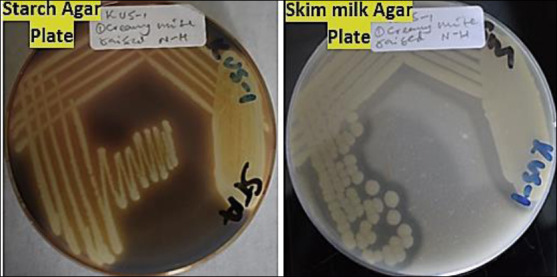
Enzymatic activities of the tested bacterial isolates showing (a) proteolytic activity, evidenced by clear zones surrounding colonies on skim milk agar, and (b) amylolytic activity, indicated by clear zones around colonies following iodine staining on starch agar (Author documentation, 2023).

Proteolytic and amylolytic activities of the selected isolates are presented in Figure 2. Among the 10 isolates screened, 40% (4/10) demonstrated protease activity, while 70% (7/10) exhibited amylase activity. Only four isolates (C, E, H, and I) expressed both enzymes.

**Figure 3:**
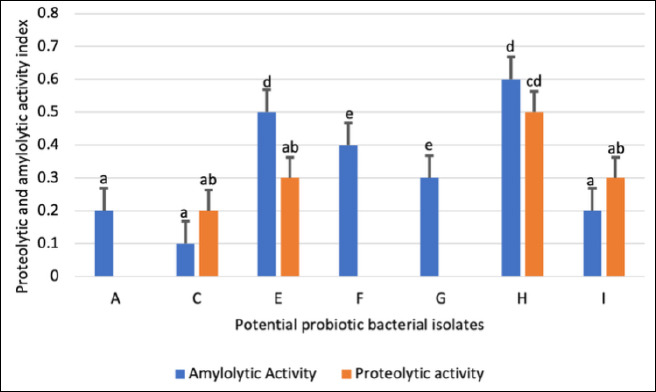
Proteolytic and amylolytic indices of potential probiotic bacterial isolates (A–J) obtained from the digestive tract of *Oreochromis niloticus*. Each bar represents the mean enzymatic index ± standard deviation (n = 3). Bars sharing the same letter are not significantly different (p < 0.05).

Isolate H exhibited the highest enzymatic indices, with proteolytic and amylolytic values of 0.5 and 0.6, respectively (Figure 3). Isolates A, F, and G showed amylolytic activity only, with isolate G presenting the highest amylase index. In contrast, isolates B, D, and J lacked both enzymatic activities and were excluded from further evaluation. Consequently, seven isolates (A, C, E, F, G, H, and I) were retained for subsequent assays.

### Bile salt tolerance

**Figure 4:**
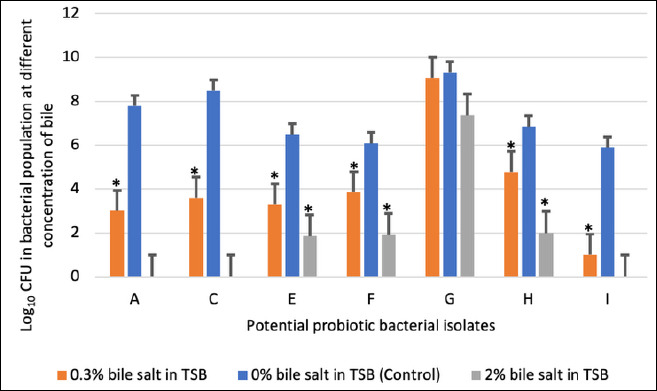
Viable counts expressed as logarithmic colony-forming units of potential probiotic bacterial isolates exposed to bile salts at concentrations of 0%, 0.3%, and 2%. Each bar represents the mean ± standard deviation (n = 3). p < 0.05 indicates a significant difference compared with the control.

**Figure 5:**
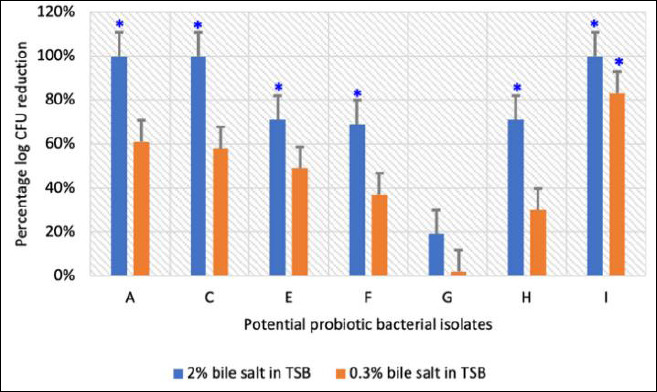
Percentage reduction in logarithmic colony-forming units of potential probiotic bacterial isolates following exposure to 0.3% and 2% bile salts. Each bar represents the mean percentage ± standard deviation (n = 3). p < 0.05 indicates a significant difference compared with the control (0% bile salts in tryptic soy broth).

Survival of the selected isolates under bile salt stress is illustrated in Figures 4 and 5. All isolates (7/7) survived in 0.3% bile salts, whereas only 57% (4/7) remained viable in 2% bile salts.

Isolates A, C, and I showed complete growth inhibition at 2% bile concentration, indicating bile intolerance. In contrast, isolates G and H exhibited minimal reductions in viable counts at both bile concentrations relative to the control, demonstrating strong bile salt tolerance (Figure 5).

### Effect of pH and exposure time on viable counts

The acid tolerance of four selected probiotic candidates (E, F, G, and H) was evaluated under simulated gastrointestinal conditions (Table 2). At baseline (0 h), viable counts differed significantly among isolates across all pH levels (p < 0.05).

**Table 2:** Survival of probiotic isolates (E–H) over time under simulated gastrointestinal pH conditions (log_10_ colony-forming units/mL).

Isolate	0 h (pH 1.5)	0 h (pH 3.0)	0 h (pH 7.2)	1.5 h (pH 1.5)	1.5 h (pH 3.0)	1.5 h (pH 7.2)	3 h (pH 1.5)	3 h (pH 3.0)	3 h (pH 7.2)
E	2.6 ± 0.60^b^	5.2 ± 0.80^b^*	5.9 ± 0.36^b^*	1.3 ± 0.26^b^	2.6 ± 0.21^b^*	5.3 ± 0.80^b^*	0.9 ± 0.15^b^	2.0 ± 0.30^b^*	4.9 ± 0.40^b^*
F	2.3 ± 0.15^b^	4.6 ± 0.53^b^*	5.3 ± 0.40^b^*	0.9 ± 0.07^c^	1.3 ± 0.21^c^*	5.0 ± 0.20^b^*	0.0 ± 0.00^b^	0.0 ± 0.00^b^	4.9 ± 0.36^b^*
G	3.2 ± 0.10^b^	6.4 ± 0.60^a^*	7.0 ± 0.56^a^*	1.8 ± 0.26^a^	3.9 ± 0.40^a^*	6.4 ± 0.30^a^*	1.2 ± 0.30^a^	3.0 ± 1.00^a^*	5.9 ± 0.21^a^*
H	2.3 ± 0.21^b^	4.8 ± 0.26^b^*	5.9 ± 0.26^b^*	1.3 ± 0.15^b^	3.0 ± 0.20^b^*	4.6 ± 0.53^b^*	0.9 ± 0.20^b^	2.0 ± 0.11^b^*	5.9 ± 0.30^a^*

Values represent mean ± standard deviation (n = 3). Different letters indicate significant differences among isolates within the same pH level at the same exposure time as determined by one-way analysis of variance followed by Tukey’s post-hoc test. Within each isolate and time point, asterisks (*) indicate values that differ significantly from at least one other pH level (p < 0.05).

Extreme acidity (pH 1.5) resulted in immediate reductions in viability compared with pH 3.0 and 7.2. Isolate G consistently exhibited the highest survival at all pH values, whereas isolate F showed the lowest tolerance. After 1.5 h of exposure, viable counts declined significantly at both acidic conditions (p < 0.05), with isolate G remaining the most acid-tolerant.

Following 3 h of exposure, further viability losses were observed. Isolate G retained the highest survival at pH 1.5 and 3.0, while isolate F exhibited complete loss of viability at both acidic levels. At pH 7.2, isolates G and H had significantly higher viable counts than E and F (p < 0.05).

### Bacterial adhesion capacity

**Figure 6:**
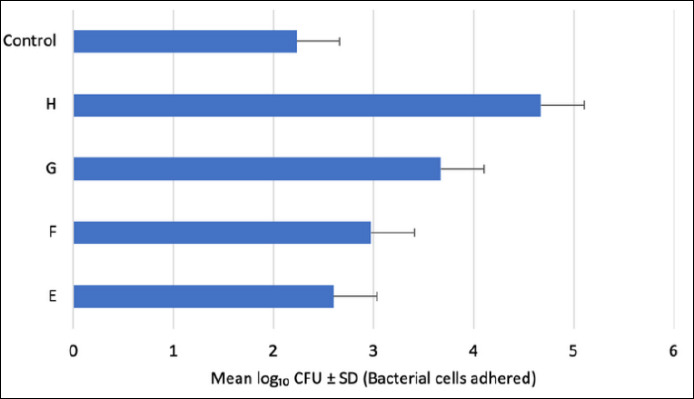
Adhesion capacity of potential probiotic bacterial isolates E, F, G, and H on stainless steel surfaces. Each bar represents the mean ± standard deviation (n = 3). No significant differences were observed between the control (*Lactiplantibacillus plantarum* ATCC 14917) and the tested isolates (E–H) (p > 0.05).

All four tested isolates demonstrated the ability to adhere to stainless steel surfaces (Figure 6). Isolate H exhibited the highest adhesion capacity (approximately 4.7 × 10^4^ CFU/mL), indicating strong colonization potential.

Isolate G also showed substantial adhesion, while isolate F exhibited moderate adherence. In contrast, isolate E demonstrated the lowest adhesion among the four candidates.

### Bacterial growth kinetics

**Figure 7:**
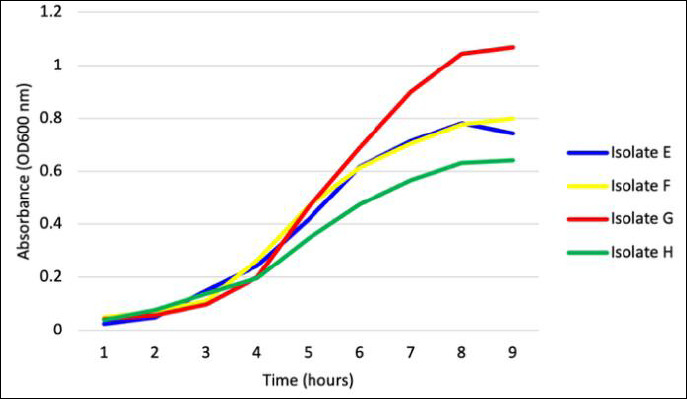
Growth curves of the four potential probiotic bacterial isolates (E–H). Data represent the mean absorbance ± standard deviation (n = 3) measured as optical density at 600 nm.

Growth patterns of all candidate isolates followed typical bacterial population dynamics, comprising lag, exponential, and stationary phases (Figure 7). The exponential growth phase commenced at approximately 3 h for all isolates, although growth intensities varied.

Isolate G demonstrated the most vigorous and prolonged exponential growth, achieving the highest optical density values. Conversely, isolate E showed the least robust growth, reaching the stationary phase earlier and at lower optical density. By 8–9 h, most isolates transitioned into the stationary phase, likely due to nutrient depletion or accumulation of metabolic by-products.

### Pathogenicity assessment in *O. niloticus*

Mortality was observed among fish inoculated with isolate G (80%), isolate E (10%), and PBS control (20%) (Table 3). No abnormal clinical signs were noted in fish challenged with isolate E or in the control group.

**Table 3:** Cumulative mortality of *Oreochromis niloticus* challenged with potential probiotic bacterial isolates over a 10-day observation period.

Treatment	Number of fish	Day 1	Day 2	Day 3	Day 4	Day 5	Day 6	Day 7	Day 8	Day 9	Day 10	Total deaths	% Mortality
Control (sterile PBS)	30	0	0	2	2	2	0	0	0	0	0	6	20%
Isolate E	30	0	1	0	1	0	1	0	0	0	0	3	10%
Isolate F	30	0	0	0	0	0	0	0	0	0	0	0	0%
Isolate G	30	0	6	0	12	0	6	0	0	0	0	24	80%
Isolate H	30	0	0	0	0	0	0	0	0	0	0	0	0%

PBS = phosphate-buffered saline

**Figure 8:**
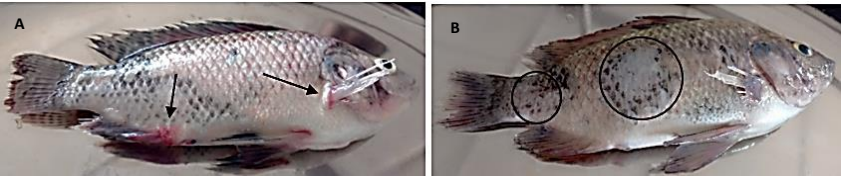
Gross pathological lesions observed in *Oreochromis niloticus* exposed to isolate G showing (A) congestion around the fin bases and operculum (black arrows) and (B) scale sloughing (black circle) (Author documentation, 2023).

In contrast, fish exposed to isolate G exhibited pronounced clinical manifestations, including lethargy, weakness, stagnation near the aquarium surface, scale desquamation, hydronephrosis, and congestion, particularly at the fin bases (Figure 8).

Mixed bacterial cultures resembling isolate G were consistently re-isolated from the kidneys and spleens of moribund and dead fish. No pathogenic bacteria were recovered from fish injected with isolate E or PBS, confirming that mortality was attributable to isolate G. These results indicated that isolates E, F, and H were non-pathogenic and suitable for further probiotic evaluation, whereas isolate G was pathogenic and excluded.

### Identification of candidate probiotic bacteria

**Figure 9:**
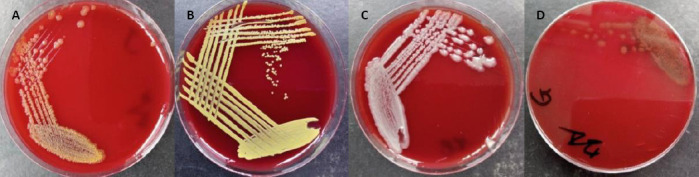
Panels illustrating colony morphology of non-pathogenic potential probiotic bacterial (A) isolates E showing pale-yellow colonies, (B) isolate F with golden-yellow pigmented colonies, (C) isolate H forming grayish-white slightly mucoid colonies, and (D) pathogenic isolate G exhibiting alpha-hemolytic creamy-white colonies (Author documentation, 2023).

Morphological, biochemical, and MALDI-TOF MS analyses were used to identify the probiotic candidates and the pathogenic isolate (Table 4; Figure 9). The three non-pathogenic probiotic isolates were identified as *R. marisflavi* (E), *M. luteus* (F), and *P. vaccinostercus* (H). The pathogenic isolate was identified as *A. ichthiosmia* (G).

**Table 4:** Phenotypic, biochemical, and MALDI-TOF MS identification of bacterial isolates obtained from the gut of *Oreochromis niloticus.*

Property	E	F	G	H
Probiotic potential	Positive	Positive	Negative	Positive
Colony morphology	Pale-yellow colonies	Circular, entire, convex, smooth, shiny, golden-yellow pigmented colonies	Creamy-white circular, smooth, and convex colonies	Grayish-white slightly mucoid colonies
Gram stain	Positive	Positive	Negative	Positive
Catalase activity	Positive	Positive	Positive	Negative
Oxidase	Negative	Positive	Positive	Negative
Indole production	Negative	Negative	Positive	Negative
Methyl red test	Positive	Negative	Negative	Positive
Citrate utilization	Negative	Negative	Positive	Negative
Urea	Negative	Positive	Negative	Negative
Triple sugar iron test	Acid butt and alkaline slant	Alkaline slant and alkaline butt	Acid slant, acid butt with gas	Alkaline slant and alkaline butt
Glucose	Positive	Negative	Positive with gas production	Negative
Sucrose	Negative	Negative	Positive with gas	Negative
Mannitol	Positive	Negative	Positive with gas	Negative
Blood hemolysis	Non-hemolytic	Non-hemolytic	Partially hemolytic	Non-hemolytic
MALDI-TOF MS score	2.301	2.376	1.866	2.128
Identity	*Rossellomorea marisflavi*	*Micrococcus luteus*	*Aeromonas ichthiosmia*	*Paucilactobacillus vaccinostercus*

MALDI-TOF MS = Matrix-assisted laser desorption/ionization time-of-flight mass spectrometry, Positive = Presence of the characteristic, Negative = Absence of the characteristic, ** = Non-pathogenic potential probiotic bacteria, * = Pathogenic bacteria.

## DISCUSSION

### Rigorous selection and novelty of indigenous probiotic isolates

Among the 50 gut bacterial isolates recovered from wild *O. niloticus*, only 10 (20%) fulfilled the preliminary probiotic screening criteria of γ-hemolysis and broad antibiotic susceptibility. Of these, seven progressed to advanced functional assays, and only three isolates (E, F, and H) successfully passed all enzymatic activity, acid–bile tolerance, adhesion, growth kinetics, and pathogenicity evaluations.

This progressive reduction from 50 initial candidates to three validated strains highlights the stringent nature of the screening protocol and underscores the scarcity of probiotic-grade autochthonous bacteria within the gut microbiota of wild tilapia. Notably, this study represents the first comprehensive isolation and probiotic screening of indigenous gut bacteria from wild *O. niloticus* inhabiting Lake Naivasha, thereby filling a critical knowledge gap left by earlier Kenyan studies that primarily focused on pond-reared fish or commercial probiotic products.

By targeting wild fish populations, the study provides novel insights into naturally adapted microbial communities that may exhibit enhanced colonization efficiency, competitive exclusion, and physiological compatibility within local aquaculture environments.

### Identification of rare probiotic species and taxonomic significance

The present study identified *R. marisflavi*, *M. luteus*, and *P. vaccinostercus* as potential probiotic candidates from the gut of wild tilapia. These bacterial species are rarely reported in fish gastrointestinal ecosystems.

Notably, *P. vaccinostercus* has not previously been described as a probiotic organism from any aquatic species. Its recent taxonomic reclassification within the genus *Paucilactobacillus* further emphasizes the limited genomic and functional information currently available for this species. The high-confidence species-level identification achieved by MALDI-TOF MS therefore constitutes the first report of *P. vaccinostercus* as a non-pathogenic, functionally promising probiotic strain in tilapia.

Similarly, *R. marisflavi* represents an underexplored bacterial taxon with considerable probiotic potential. Together, these isolates constitute valuable microbial resources for future probiotic development. However, molecular verification remains essential to confirm their safety profiles, particularly given reports of opportunistic pathogenicity among closely related strains. Depositing these isolates in accredited microbial repositories is strongly recommended to facilitate downstream genomic analyses and formulation research.

### Functional probiotic traits in relation to previous studies

Although no prior studies have specifically reported *R. marisflavi*, *M. luteus*, or *P. vaccinostercus* as probiotics in *O. niloticus*, the functional traits observed in the present isolates align closely with those documented for other probiotic genera isolated from fish gastrointestinal tracts.

Previous investigations have demonstrated that probiotic bacteria from *O. niloticus* commonly exhibit enzymatic activity, bile and acid tolerance, and adhesion capacity—features that collectively enhance nutrient digestion, gut health, and disease resistance [21]. Reda *et al*. [40] and Athulya *et al*. [47] similarly reported substantial enzymatic and antimicrobial activities among gut-derived isolates, including *Lactococcus lactis*, *Enterococcus faecalis*, *Lysinibacillus* spp., *Citrobacter freundii*, and *Bacillus* spp., thereby reinforcing their probiotic suitability.

Adhesion to intestinal surfaces plays a crucial role in microbial persistence, pathogen exclusion, and immune stimulation [48]. According to Torres-Maravilla *et al*. [49], adhesive probiotics enhance mucosal immunity and epithelial barrier integrity, enabling sustained probiotic colonization and protective host responses.

Effective probiotic candidates must also withstand gastrointestinal stresses, including acidic pH and elevated bile concentrations. The physiological bile concentration in fish intestines ranges from 0.4% to 1.3%, while experimental screening commonly employs concentrations between 2.5% and 10% [50]. In the present study, isolate H exhibited superior tolerance to both acidic and bile environments over prolonged exposure periods, suggesting high probiotic suitability.

Comparable findings were reported by Balcázar *et al*. [50] and Coulibaly *et al*. [51], who isolated LAB from *O. niloticus* that demonstrated strong acid–bile tolerance, adhesion capacity, and antagonistic activity against fish pathogens. Iorizzo *et al*. [52] further confirmed these probiotic traits in *Lactiplantibacillus plantarum* derived from trout intestines.

Unlike many earlier screening studies that relied solely on *in vitro* assays, the current investigation uniquely incorporated *in vivo* pathogenicity testing to ensure host safety, consistent with the Food and Agriculture Organization (FAO) probiotic evaluation guidelines. Collectively, the results affirm the gut microbiome of *O. niloticus* as a rich reservoir of safe indigenous bacteria with multifunctional probiotic potential.

The observed antagonistic activity also aligns with global evidence that probiotics produce antimicrobial compounds, such as organic acids, bacteriocins, siderophores, and lipopeptides, that inhibit common aquaculture pathogens, including *Aeromonas*, *Vibrio*, and *Streptococcus* species [53].

### Probiotic potential and emerging importance of *P. vaccinostercus*

Probiotic strains exhibiting characteristics similar to those observed in this study have previously been isolated from the gastrointestinal tract of *O. niloticus* [54]. Meidong *et al*. [55] further demonstrated that *L. plantarum* showed strong acid–bile tolerance, adhesion capacity, and non-hemolytic behavior.

In contrast to commonly used probiotic genera such as *Bacillus* and *Lactobacillus*, *P. vaccinostercus* displayed comparable acid and bile resistance, along with superior adhesion potential, suggesting high adaptation to the tilapia gut environment.

*P. vaccinostercus*, recently reclassified within the *Paucilactobacillus* clade, is recognized for its metabolic versatility and aerotolerance—traits that support probiotic functionality. Genomic studies have identified genes associated with vitamin biosynthesis, stress tolerance, and antimicrobial activity in *Paucilactobacillus* species, suggesting roles in nutrient assimilation, pathogen exclusion, and immune modulation [53, 56].

Despite the established probiotic use of LAB in aquaculture, strain-level characterization of *P. vaccinostercus* remains limited, and rigorous safety assessment, including screening for transferable AMR genes, is essential before application [49].

When benchmarked against commercial probiotics such as *Bacillus* spp. and *L. plantarum*, the isolates identified in this study, particularly *P. vaccinostercus*, demonstrated comparable or superior performance across acid tolerance, bile resistance, enzymatic activity, and adhesion. This suggests that locally adapted strains may offer enhanced ecological compatibility, cost efficiency, and sustainability for Kenyan aquaculture systems.

### Probiotic mechanisms and performance of *R. marisflavi*

*R. marisflavi*, formerly classified as *Bacillus marisflavi*, belongs to the family *Bacillaceae* within the phylum *Firmicutes* and was reassigned to the genus *Rossellomorea* based on phylogenomic evidence [57, 58].

Recent experimental studies, including *in vivo* trials in *O. niloticus*, have shown that dietary supplementation with *R. marisflavi*, often in probiotic consortia, can improve growth performance, feed utilization efficiency, survival under bacterial challenge, and immune parameters [59].

The probiotic effects of *R. marisflavi* are attributed to multiple mechanisms, including competitive exclusion of pathogens, secretion of digestive enzymes (amylases, proteases, lipases), and production of antimicrobial metabolites [57]. The present study confirmed several of these probiotic attributes under *in vitro* conditions.

### Strain-specific probiotic and pathogenic characteristics of *M. luteus*

The current findings demonstrated that *M. luteus* exhibits multiple probiotic-associated traits, including antagonism against fish pathogens, extracellular enzyme production, acid–bile tolerance, adhesion capacity, and favorable antibiotic susceptibility profiles.

Previous experimental studies have further reported growth-promoting and protective effects of *M. luteus* in *O. niloticus* under pathogenic challenge, supporting its probiotic candidacy [59].

However, recent evidence also highlights the strain-specific nature of *M. luteus*. A 2025 study from India identified pathogenic *M. luteus* strains causing severe disease and high mortality in farmed tilapia [60]. These contrasting outcomes emphasize the importance of comprehensive safety evaluation, including virulence assessment and AMR profiling, before probiotic application.

### Pathogenic nature of *A. ichthiosmia* and safety implications

The present study also isolated *A. ichthiosmia* from the gut of *O. niloticus*. Although this isolate exhibited several probiotic-like traits, including enzymatic activity, acid–bile tolerance, and adhesion capacity, it was ultimately confirmed as pathogenic.

The experimental challenge fulfilled Koch’s postulates, with affected fish exhibiting lethargy, congestion at the fin bases, mortality, and re-isolation of the same bacterial strain from internal organs.

Given the limited prior documentation of *A. ichthiosmia* pathogenicity in fish, these findings represent one of the first well-characterized reports identifying this species as a fish pathogen. This underscores the critical necessity of *in vivo* safety testing alongside functional probiotic screening.

### Implications for sustainable aquaculture and future research

The development of indigenous probiotic strains offers significant potential for sustainable aquaculture within Kenya’s Blue Economy framework by reducing reliance on antibiotics and mitigating AMR risks.

Utilization of native, non-pathogenic strains aligns with FAO–WOAH–WHO One Health strategies by enhancing fish health through biological mechanisms while preserving ecological balance.

The application of MALDI-TOF MS in this study demonstrated its utility for rapid, accurate bacterial identification, strengthening local diagnostic capacity. However, limitations include the absence of whole-genome sequencing, strain-level genomics, and metabolomic profiling.

Future research will integrate genomic, metabolomic, and *in vivo* performance evaluations to confirm safety, elucidate probiotic mechanisms, and support regulatory approval for commercial application.

## CONCLUSION

This study successfully isolated and rigorously screened autochthonous gut bacteria from wild *O. niloticus*, leading to the identification of three non-pathogenic probiotic candidates, namely *R. marisflavi*, *M. luteus*, and *P. vaccinostercus*. Of 50 initial isolates, only 10 met the preliminary safety and antibiotic-susceptibility criteria; 7 advanced to functional assays, and ultimately 3 strains passed all enzymatic activity, acid–bile tolerance, adhesion, growth kinetics, and *in vivo* pathogenicity evaluations. These findings demonstrate the rarity of probiotic-grade indigenous bacteria in the gut microbiome of wild tilapia and underscore the importance of stringent multistage screening approaches.

Functionally, the selected isolates exhibited strong probiotic attributes, including extracellular enzyme production, high tolerance to gastrointestinal stress conditions, effective surface adhesion, and antagonistic activity against common fish pathogens. Among the candidates, *P. vaccinostercus* displayed particularly superior performance in acid–bile resistance and adhesion potential, highlighting its high adaptability to the tilapia gut environment. Importantly, this study provides the first documented evidence that *P. vaccinostercus* is a safe and promising probiotic in tilapia, thereby expanding the diversity of probiotic taxa applicable to aquaculture.

From a practical perspective, the utilization of these indigenous probiotic strains holds substantial promise for improving fish health, enhancing feed utilization efficiency, and reducing reliance on antibiotics within Kenyan aquaculture systems. The ecological compatibility of locally adapted strains offers advantages in terms of colonization efficiency, sustainability, cost-effectiveness, and minimized environmental disruption. These findings align with One Health strategies aimed at mitigating the emergence of AMR while promoting biologically based disease control in aquaculture.

A major strength of this study lies in its comprehensive screening framework, which integrated functional *in vitro* assays with *in vivo* pathogenicity testing to ensure both efficacy and host safety. The application of MALDI-TOF MS enabled rapid and accurate species-level identification, representing a significant methodological advancement within Kenyan fish microbiology research. Furthermore, the focus on wild fish populations provided novel insights into naturally adapted gut microbiota that are often overlooked in probiotic development studies.

Nevertheless, the study has certain limitations. The absence of whole-genome sequencing and strain-level comparative genomics restricts a deeper understanding of probiotic mechanisms, virulence potential, and AMR gene profiles. Additionally, metabolomic analyses were not performed to characterize bioactive compounds responsible for antagonistic effects. The probiotic performance of the isolates was not evaluated under commercial farming conditions, which may influence their functional efficacy.

Future research should prioritize genome-based safety assessment, functional gene annotation, and metabolite profiling of the identified probiotic strains. Large-scale *in vivo* feeding trials are warranted to evaluate growth performance, immune modulation, disease resistance, and long-term colonization in cultured tilapia. Moreover, formulation studies focusing on delivery methods, dosage optimization, and shelf stability will be critical for successful commercial application.

In conclusion, the gut microbiome of wild *O. niloticus* represents a valuable reservoir of indigenous probiotic bacteria with strong functional potential. The identification of *R. marisflavi*, *M. luteus*, and especially the novel candidate *P. vaccinostercus* provides a scientific foundation for the development of locally adapted probiotic solutions for sustainable aquaculture. With further genomic validation and applied trials, these strains could significantly enhance fish health, productivity, and antimicrobial stewardship in the aquaculture sector.

## DATA AVAILABILITY

The raw datasets (plate counts, OD readings, and MALDI spectra) generated and analyzed during the study are available upon reasonable request from the corresponding author.

## AUTHORS’ CONTRIBUTIONS

RDK, DWW, JJNN, and PNN: Planned and designed the study. JJNN and PNN: Supervised the study, data analysis and interpretation, and revised the manuscript. RDK and DWW: Performed the field and laboratory work and drafted the manuscript. DWW: Analyzed the data. All authors have read and approved the final version of the manuscript.
